# Supplementary material for: Significance of MnO2 Type and Solution Parameters in Manganese Removal from Water Solution
Source: Int J Mol Sci. 2023 Feb 23;24(5):4448. doi: 10.3390/ijms24054448 (PMC10003147; doi:10.3390/ijms24054448)
Supplement: Supplementary file 1 [file ijms-24-04448-s001.zip › ijms-2203452-supplementary/Supplementary Materials.pdf]

<u>SUPPLEMENTARY MATERIALS</u>

Significance of MnO_2 type and solution parameters in manganese removal from water solution

Magdalena M. Michel [1], Mostafa Azizi [1,*], Dorota Mirosław-Świątek [1], Lidia Reczek [1], Bogumił Cieniek [2], and Eleonora Sočo [3]

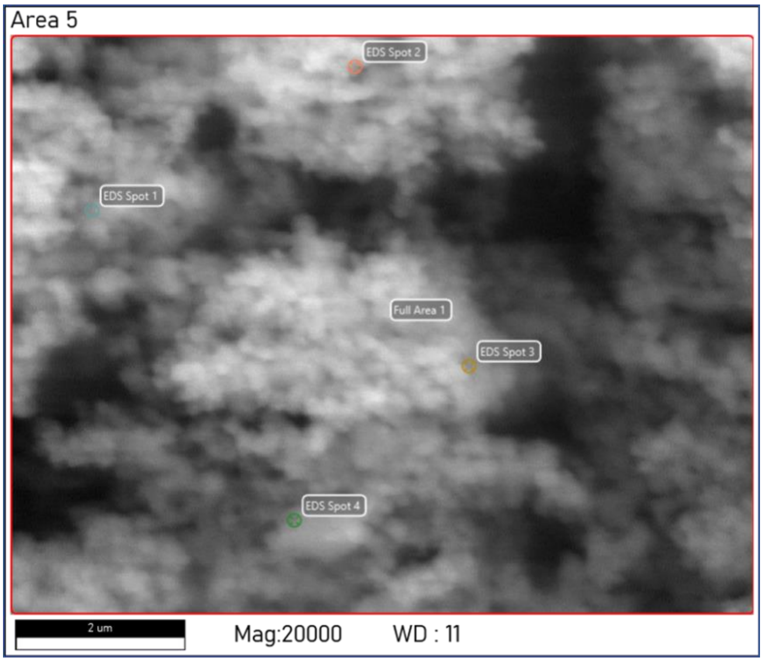

(**a**)

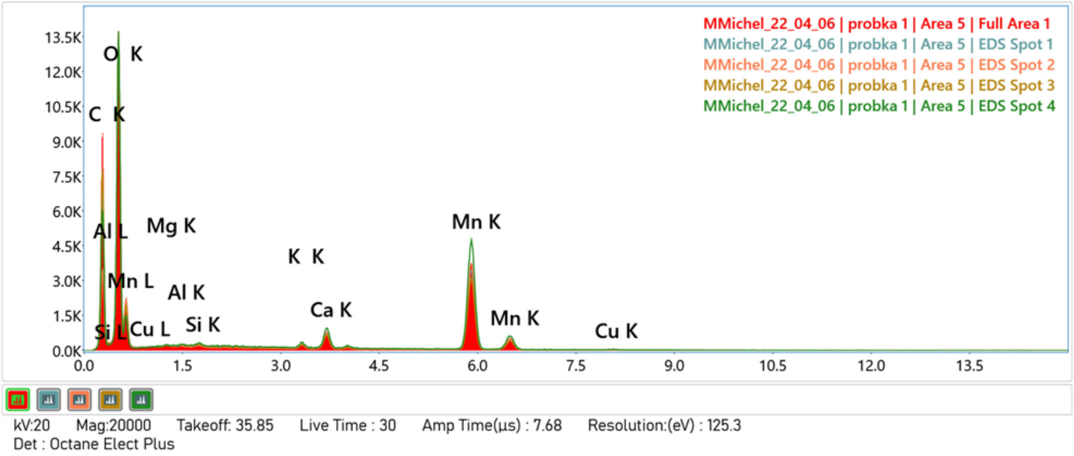

(**b**)

Figure S1. SEM-EDS analysis of AKH surface: (**a**) The location of the measurement spots 1–4; (**b**) The spectra obtained from the measurement spots 1–4.

Table S1. Element composition of AKH surface, atomic %.

Element	Spot 1	Spot 2	Spot 3	Spot 4
C	36.9	17.8	39.8	40.5
O	49.1	56.4	50.4	47.8
Mn	11.7	21.9	8.4	10.1
Mg	0.3	0.3	0.1	0.1
Al	0.1	0.2	0.1	n.d.
Si	0.1	0.2	0.1	0.1
K	0.3	0.6	0.2	0.2
Cu	0.1	n.d.	n.d.	n.d.
Ca	1.3	2.6	1.0	1.1

n.d. – not detected.

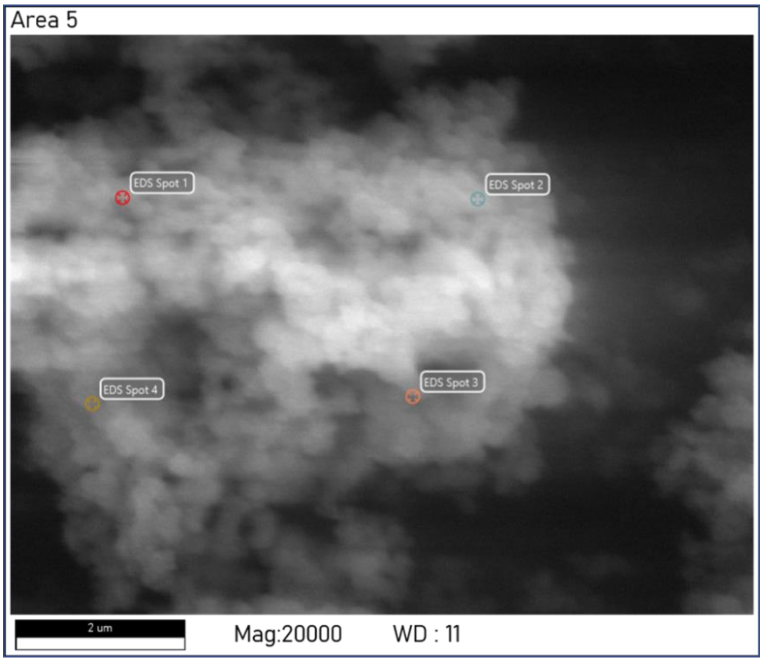

(**a**)

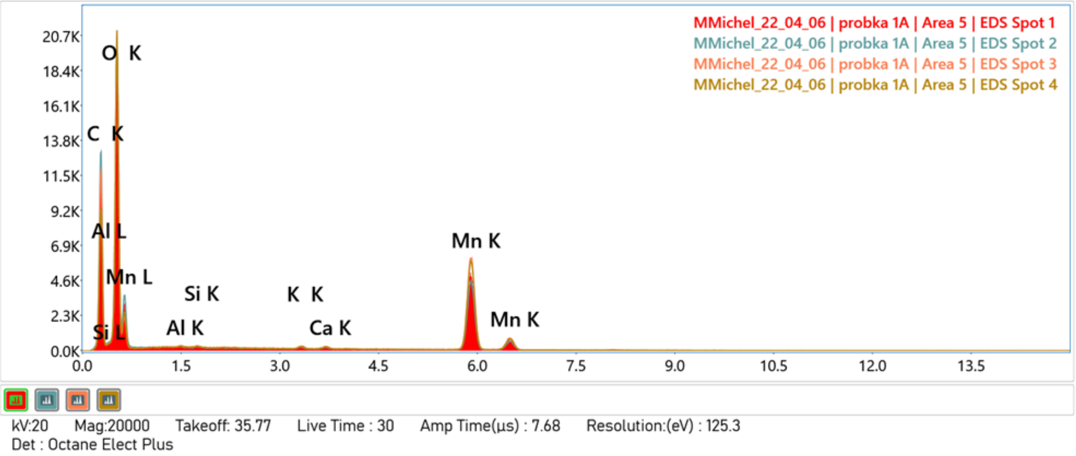

(**b**)

Figure S2. SEM-EDS analysis of AKH surface after Mn adsorption: (**a**) The location of the measurement spots 1–4; (**b**) The spectra obtained from the measurement spots 1–4.

Table S2. Element composition of AKH surface after Mn adsorption, atomic %.

Element	Spot 1	Spot 2	Spot 3	Spot 4
C	32.6	42.6	39.2	38.9
O	59.9	52.7	53.9	54.2
Mn	7.0	4.4	6.6	6.5
Al	0.2	0.1	0.1	0.1
Si	0.1	n.d.	n.d.	0.1
K	0.1	0.1	0.1	0.1
Ca	0.1	0.1	0.1	0.1

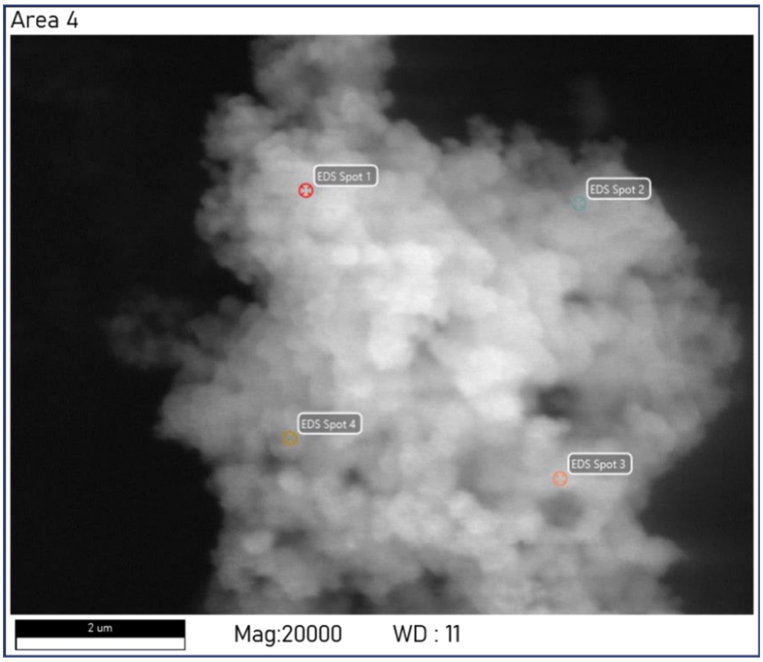

(**a**)

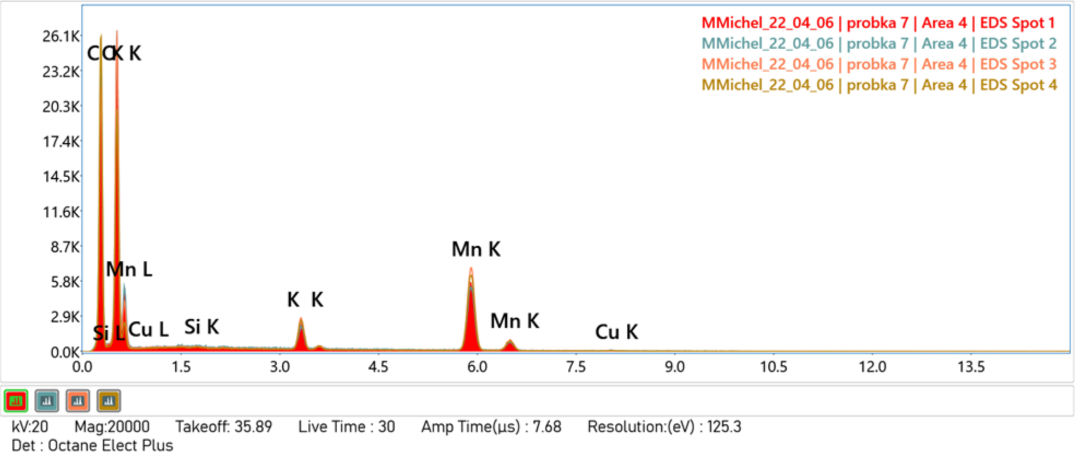

(**b**)

Figure S3. SEM-EDS analysis of BIR surface: (**a**) The location of the measurement spots 1–4; (**b**) The spectra obtained from the measurement spots 1–4.

Table S3. Element composition of BIR surface, atomic %.

Element	Spot 1	Spot 2	Spot 3	Spot 4
C	52.6	55.1	53.2	60.4
O	44.0	42.0	42.8	35.9
Mn	2.8	2.4	3.3	3.0
K	0.6	0.4	0.7	0.6
Cu	n.d.	0.1	n.d.	n.d.

n.d. – not detected.

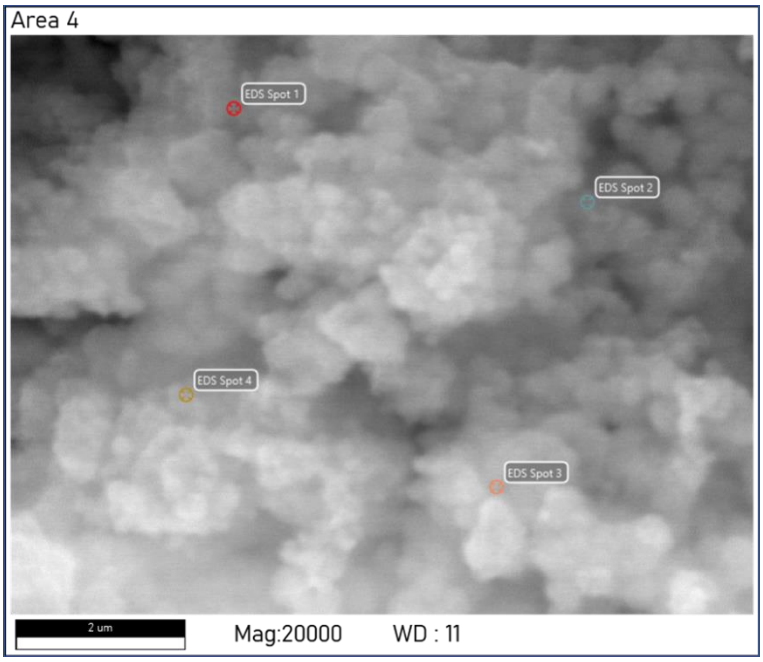

(**a**)

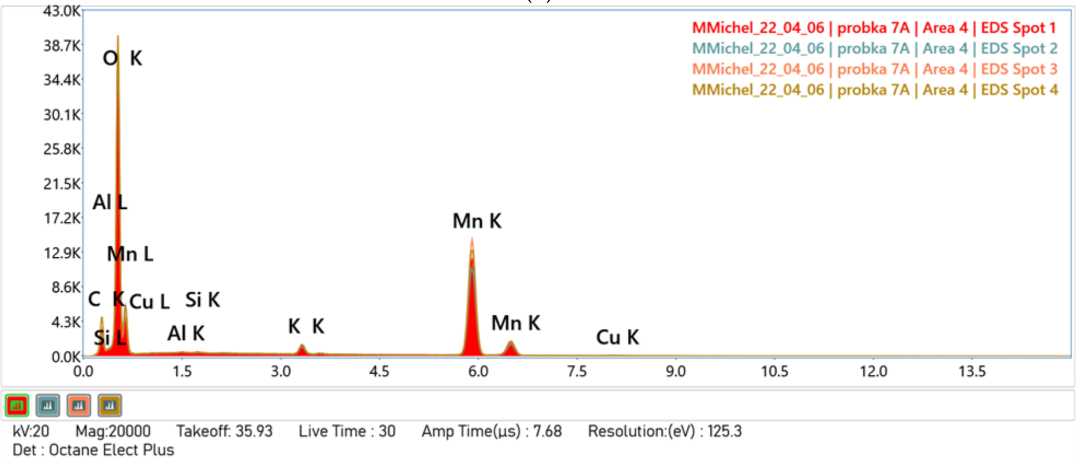

(**b**)

Figure S4. SEM-EDS analysis of BIR surface after Mn adsorption: (**a**) The location of the measurement spots 1–4; (**b**) The spectra obtained from the measurement spots 1–4.

Table S4. Element composition of BIR surface after Mn adsorption, atomic %.

Element	Spot 1	Spot 2	Spot 3	Spot 4
C	13.8	8.9	11.0	22.4
O	69.6	72.9	67.0	64.0
Mn	15.6	16.6	20.3	12.6
Al	0.3	0.5	0.4	0.2
Si	n.d.	0.2	0.1	0.1
K	0.7	0.8	0.9	0.6
Cu	n.d.	0.1	0.2	0.1

n.d. – not detected.

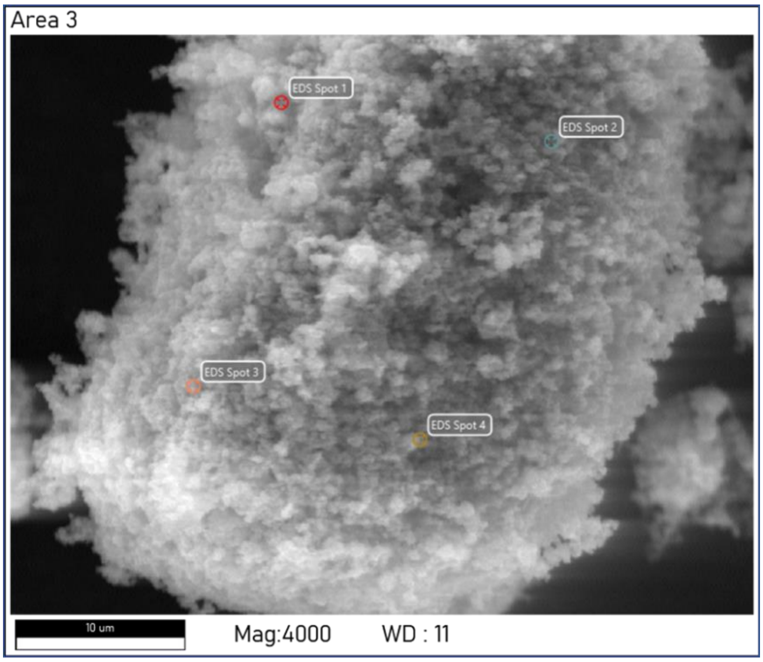

(**a**)

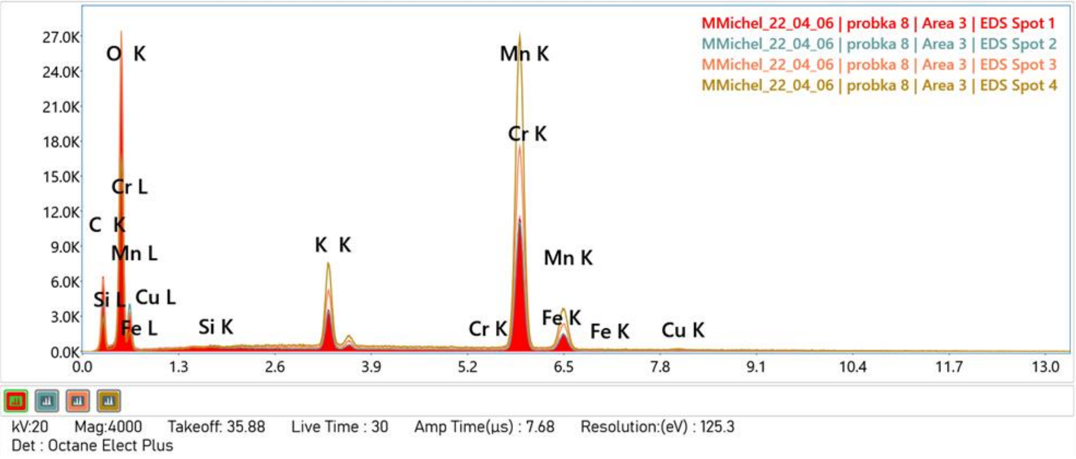

(**b**)

Figure S5. SEM-EDS analysis of CRY surface: (**a**) The location of the measurement spots 1–3; (**b**) The spectra obtained from the measurement spots 1–3.

Table S5. Element composition of CRY surface, atomic %.

Element	Spot 1	Spot 2	Spot 3
C	33.5	20.1	26.7
O	54.9	62.2	52.3
Mn	9.9	15.1	18.0
Fe	n.d.	0.1	0.2
K	1.6	2.4	2.7
Cu	0.1	0.1	0.1

n.d. – not detected.

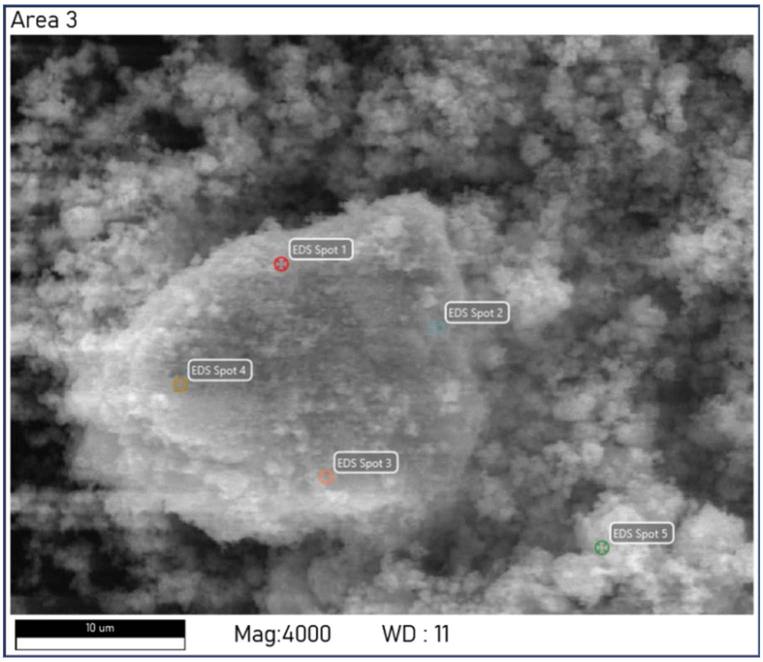

(**a**)

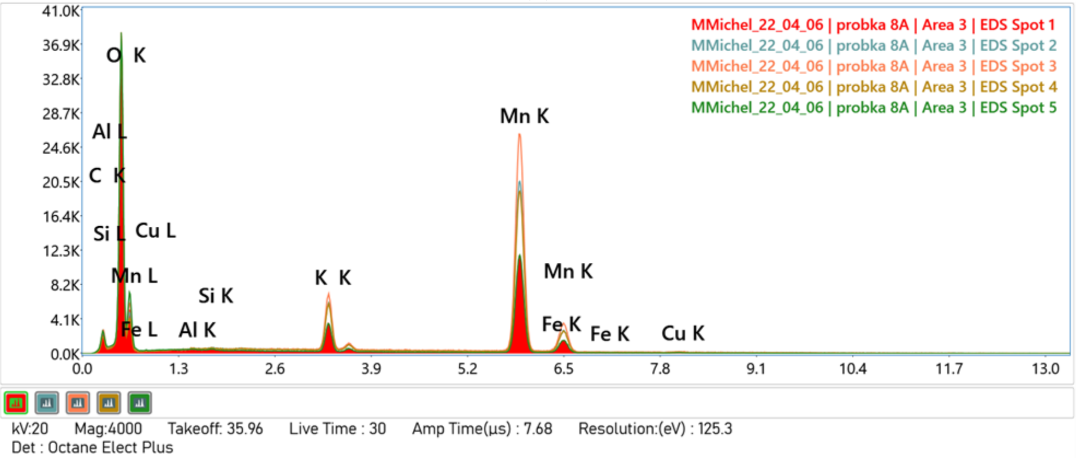

(**b**)

Figure S6. SEM-EDS analysis of CRY surface after Mn adsorption: (**a**) The location of the measurement spots 1–4; (**b**) The spectra obtained from the measurement spots 1–5.

Table S6. Element composition of CRY surface after Mn adsorption, in atomic %.

Element	Spot 1	Spot 2	Spot 3	Spot 4	Spot 5
C	12.7	7.9	12.7	9.8	10.8
O	71.1	64.5	59.8	66.6	72.7
Mn	13.6	23.8	23.9	20.4	14.1
Al	0.2	n.d.	n.d.	n.d.	n.d
Si	n.d.	n.d.	n.d.	n.d.	0.2
Fe	0.1	0.2	0.3	n.d.	n.d
K	2.1	3.5	3.2	3.1	2.1
Cu	0.1	0.1	0.2	0.2	0.1

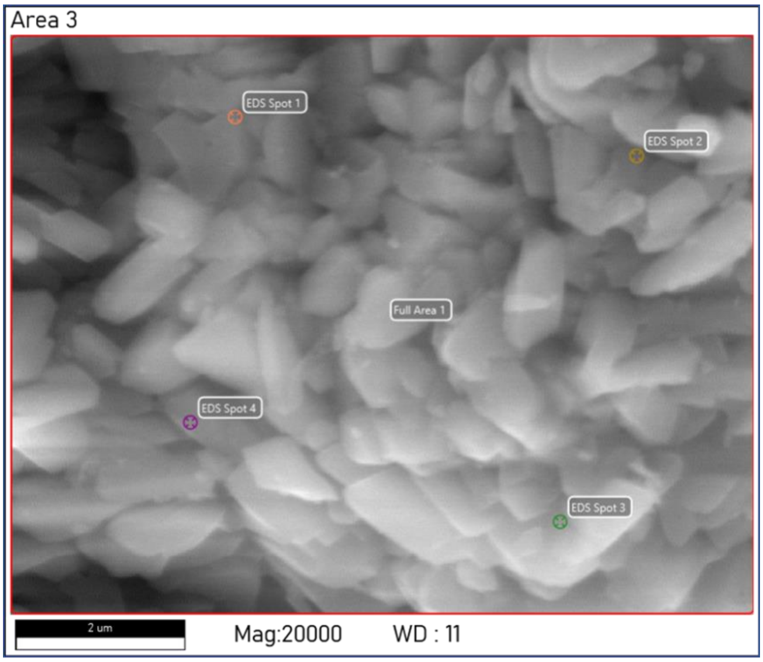

(**a**)

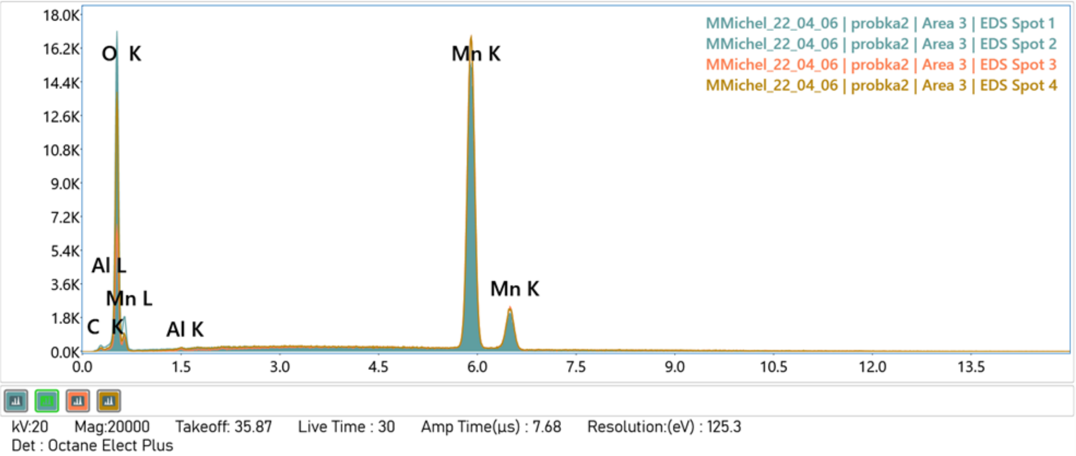

(**b**)

Figure S7. SEM-EDS analysis of PYR surface: (**a**) The location of the measurement spots 1–4; (**b**) The spectra obtained from the measurement spots 1–4.

Table S7. Element composition of PYR surface, atomic %.

Element	Spot 1	Spot 2	Spot 3	Spot 4
C	6.9	n.d.	n.d.	n.d.
O	65.5	56.9	31.9	46.9
Mn	27.5	43.1	68.1	53.1
Al	0.2	n.d.	n.d.	n.d.

n.d. – not detected.

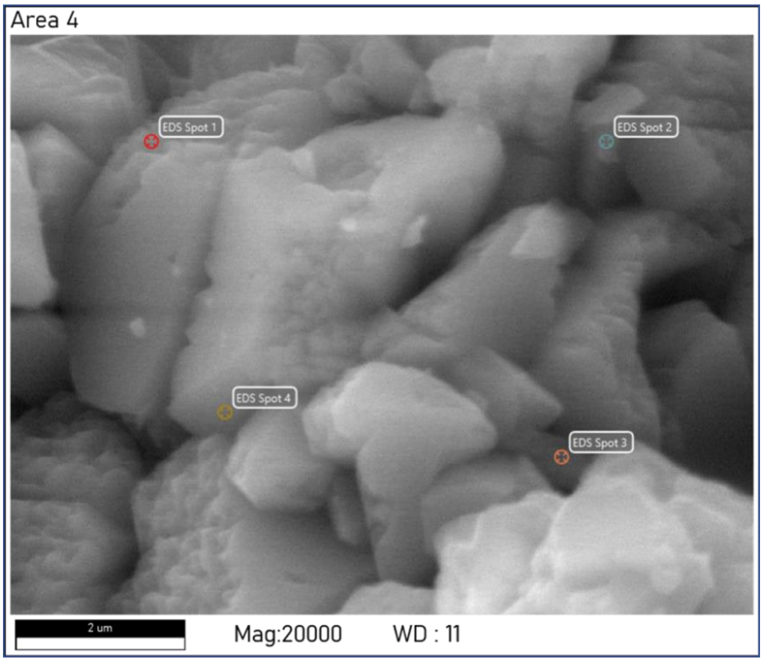

(**a**)

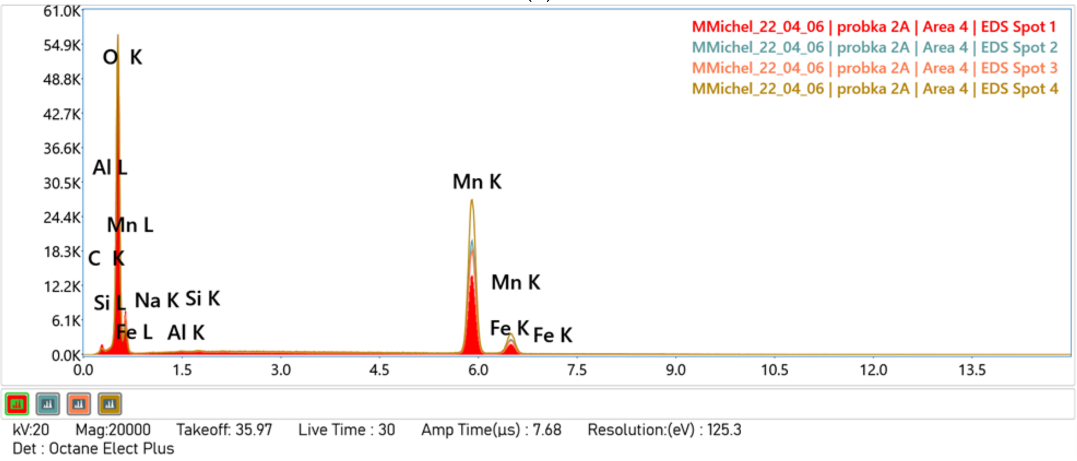

(**b**)

Figure S8. SEM-EDS analysis of PYR surface after Mn adsorption: (**a**) The location of the measurement spots 1–4; (**b**) The spectra obtained from the measurement spots 1–4.

Table S8. Element composition of PYR surface after Mn adsorption, atomic %.

Element	Spot 1	Spot 2	Spot 3	Spot 4
C	14.9	7.4	11.5	5.2
O	70.4	69.2	70.6	67.5
Mn	14.0	22.0	17.7	27.3
Al	0.4	0.2	0.2	n.d.
Si	0.2	0.1	0.1	n.d.
Na	n.d.	1.0	n.d.	n.d.
Fe	0.1	n.d.	n.d.	n.d.
